# Systemic Loss and Gain of Chromatin Architecture throughout Zebrafish Development

**DOI:** 10.1016/j.celrep.2018.06.003

**Published:** 2018-07-03

**Authors:** Lucas J.T. Kaaij, Robin H. van der Weide, René F. Ketting, Elzo de Wit

**Affiliations:** 1 Institute of Molecular Biology, 55128 Mainz, Germany; 2 Oncode Institute and Division of Gene Regulation, Netherlands Cancer Institute, Plesmanlaan 121, 1066 CX Amsterdam, the Netherlands

## Abstract

The spatial organization of chromosomes is critical in establishing gene expression programs. We generated *in situ* Hi-C maps throughout zebrafish development to gain insight into higher-order chromatin organization and dynamics. Zebrafish chromosomes segregate in active and inactive chromatin (A/B compartments), which are further organized into topologically associating domains (TADs). Zebrafish A/B compartments and TADs have genomic features similar to those of their mammalian counterparts, including evolutionary conservation and enrichment of CTCF binding sites at TAD borders. At the earliest time point, when there is no zygotic transcription, the genome is highly structured. After zygotic genome activation (ZGA), the genome loses structural features, which are re-established throughout early development. Despite the absence of structural features, we see clustering of super-enhancers in the 3D genome. Our results provide insight into vertebrate genome organization and demonstrate that the developing zebrafish embryo is a powerful model system to study the dynamics of nuclear organization.

## Highlights

• We have generated Hi-C maps throughout zebrafish development
• Zebrafish TADs and compartments have features similar to mammalian genome structures
• Genome organization is lost and gained during development
• Super-enhancers are found clustered even in the absence of compartments

## Graphical Abstract

**Figure:**
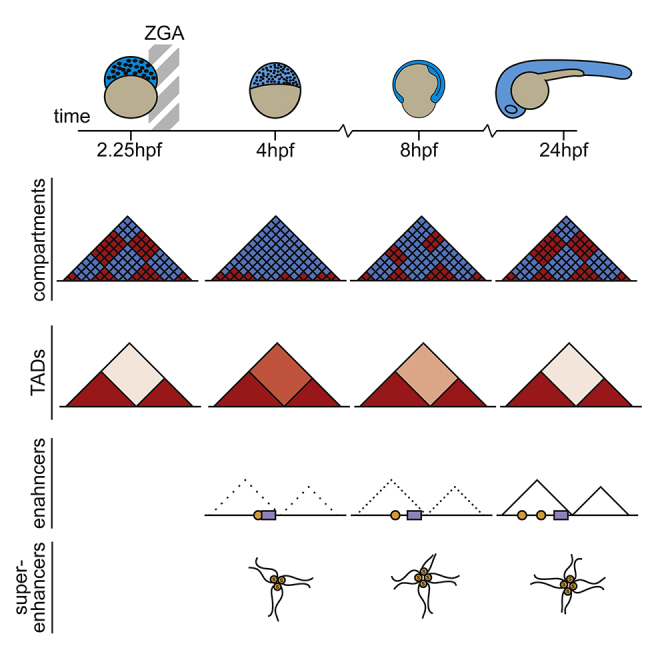

## Teaser

How do developing zebrafish embryos organize their genome? Kaaij et al. show that, early in development, when there is no transcription, the genome is highly structured; however, when the zygotic genome is activated, this organization is lost. Later in development, the genome again adopts a structured organization.

## Introduction

The spatial organization of the nucleus facilitates the interaction between distant functional elements in the genome (Tolhuis et al., 2002) and simultaneously inhibits the unwanted spatial interaction of functional elements (Dowen et al., 2014). Chromosome conformation capture (3C) studies have been instrumental in revealing the structural features of genomes (Dekker et al., 2002). For instance, Hi-C experiments have shown that interphase chromosomes are hierarchically structured (Lieberman-Aiden et al., 2009) and that this structure is lost during metaphase (Naumova et al., 2013). Chromosomes separate active and inactive chromatin into A and B compartments, respectively. The A compartment correlates with high gene expression, active histone marks, and early replication timing, whereas the B compartment is late replicating and enriched for repressive histone modifications and low gene expression.

Compartments can be further subdivided into megabase-sized genomic regions known as topologically associating domains (TADs) (Dixon et al., 2012, Nora et al., 2012), which act as regulatory scaffolds and are demarcated by binding sites of the architectural protein CTCF. Disruption of TAD boundaries results in the establishment of novel inter-TAD interactions. These have been shown to be associated with misexpression of *Hox* genes (Narendra et al., 2015), upregulation of proto-oncogenes (Flavahan et al., 2016), and developmental disorders (Lupiáñez et al., 2015). Despite the strong links between nuclear organization and gene expression, it remains unclear how TADs, loops, and compartments contribute to gene regulation, both in steady state and throughout development.

Efforts in *Drosophila* and mouse have delineated the 3D genome dynamics throughout development (Du et al., 2017, Hug et al., 2017, Ke et al., 2017). It was shown that there is a marked absence of both TADs and compartments early in mouse embryogenesis and that these structures are gradually established following zygotic genome activation (ZGA). Although TADs are largely established post-ZGA, it was shown in both mouse and fly that transcription is not required to initiate TAD formation.

In zebrafish, before ZGA, the cell cycle takes ∼15 min, does not have gap phases, and consists solely of S and M phases. Post-ZGA, the S phase lengthens and the G2 phase appears (Kimmel et al., 1995, Siefert et al., 2017). With the initiation of zygotic transcription, the zygotic dependence on maternally provided mRNAs gradually decreases and histone modifications associated with active transcription and repression appear (Bogdanovic et al., 2012, Heyn et al., 2014, Lee et al., 2014, Lindeman et al., 2011, Vastenhouw et al., 2010). Enhancer-TSS interactions are present post-ZGA in zebrafish and are often stable (Gómez-Marín et al., 2015, Kaaij et al., 2016); however, little is known about *in vivo* higher-order chromatin structures throughout development. To address this, we present multiple Hi-C datasets spanning time points before ZGA until 24 hr post fertilization (hpf), a time point at which most organs have been established.

## Results

### Zebrafish Chromosome Folding Is Consistent with Known Features of 3D Genome Organization

**Figure 1:**
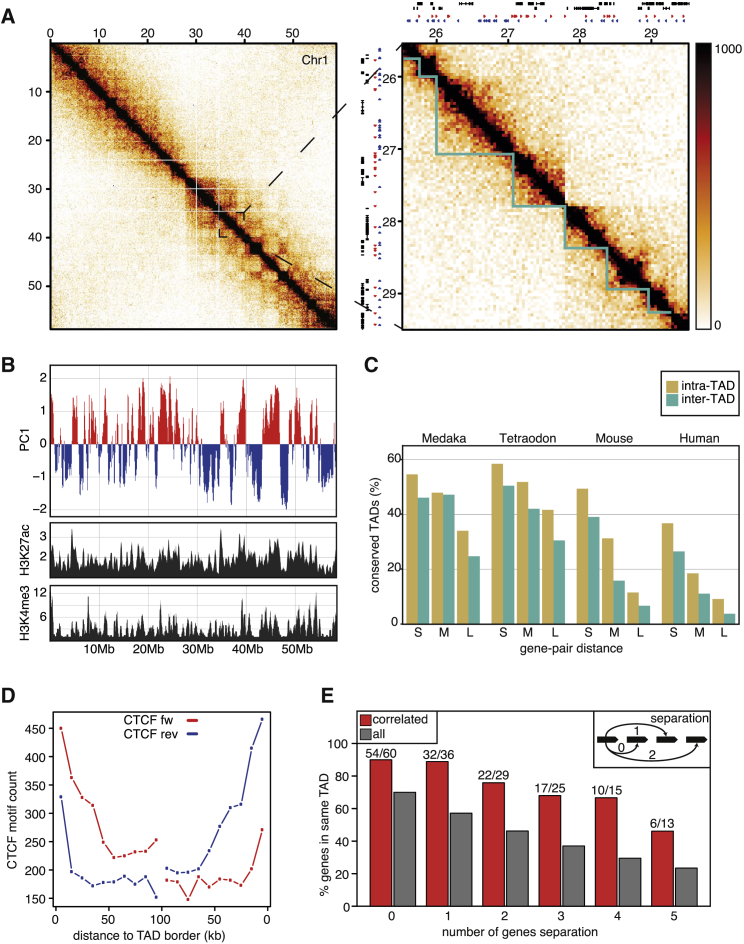
Characteristics of Zebrafish 3D Genome Organization at 24 hpf (A) Hi-C contact matrix of chromosome 1 at 40-kb resolution at 24 hpf (left panel). Zoom-in of a ∼4-Mb region of the right arm of chromosome 1 (right panel). The Hi-C contact matrix is the average of four biological replicates. Above the Hi-C contact matrix, gene models are indicated in black and inferred CTCF binding sites are displayed in red (forward) and blue (reverse) triangles. (B) Plot showing the first principal component from HOMER for chromosome 1 (upper panel). ChIP-seq tracks of H3K27ac and H3K4me3 as indicated (lower panels). (C) Plot depicting the mean intra- and inter-TAD conservation scores between zebrafish and two ray-finned fish species, as well as two mammalian species, stratified on the distance between the investigated gene pairs (100–235 kb [S, short], 235–534 kb [M, medium], and 534–1,212 kb [L, long]). (D) Motif count and orientation of inferred CTCF binding sites at 24 hpf relative to TAD borders. (E) Representative barplot of the percentage of correlated gene pairs (ρ > 0.5) based on Tomo-seq data (red bars) within the same TADs compared to all gene pairs (gray bars). Tested gene pairs are stratified based on the number of genes they are separated by, as schematically depicted (upper-right inset). The distance is indicated underneath the barplot. Fisher’s method was used to combine the p values of the binomial tests that were performed for each gene-pair distance (p < 1 × 10^−11^).

To study the 3D genome organization in zebrafish, we generated Hi-C maps of 24-hpf embryos and plotted the observed interaction frequencies as a heatmap (Figure 1A). Visual inspection revealed that the zebrafish genome at the whole-chromosome level shows compartmentalization (Lieberman-Aiden et al., 2009). We used HOMER to call A/B compartments at 100-kb resolution (Figure 1B). As in mammals, we found that A compartments are enriched for H3K4me3, H3K4me1, and H3K27ac (Figure 1B; Figure S1A). In addition, A compartments are more gene dense and show a higher level of transcription (Figures S1A and S1B). These results suggest that compartmentalization in the zebrafish genome is governed by the same biochemical principles as in mammals.

At higher resolution, it becomes apparent that the A/B compartments are further subdivided into TADs, which we identified using CatCH (Zhan et al., 2017). Visual inspection of the called TADs revealed that some TAD calls appear to be scaffolding errors. Although Hi-C data theoretically allow for re-scaffolding of chromosomes (Burton et al., 2013, Kaplan and Dekker, 2013), the resolution of our dataset does not permit this (data not shown). We therefore devised a computational strategy (STAR Methods) to identify and remove these genomic rearrangements from the TAD dataset. After a final, manual curation of the dataset, ∼1,700 TADs were identified. The median size of the TADs is ∼500 kb in zebrafish, which is within the same order of magnitude as observed in mouse and human (∼800 kb). Next, we analyzed genomic features at TAD boundaries. Similar to other organisms (Dixon et al., 2012), we found that in zebrafish, TSSs are enriched at TAD boundaries (Figure S1C). We used published RNA sequencing (RNA-seq) datasets to determine whether genes are tissue specific or broadly expressed (housekeeping) by calculating the Shannon entropy score for published RNA-seq datasets (see STAR Methods for details). We found, also in zebrafish, that housekeeping genes are enriched at TAD boundaries, whereas tissue-specific genes are only slightly enriched over background (Figure S1D).

Another characteristic of mammalian TADs is the conservation of borders in the genome. To determine the degree of conservation of zebrafish TADs, we compared the position of orthologous genes within TADs between zebrafish and two species of ray-finned fish (i.e., Medaka or Japanese rice fish, *Orizias latipes*, and green spotted pufferfish, *Tetraodon nigroviridis*), as well as two species of mammals (human and mouse). Because the positions of TAD borders for the fish species are unknown, we asked whether gene pairs that are found together in a zebrafish TAD are found within 1 Mb of each other on the same chromosome in the species we compare them to. If a TAD contains one or more conserved gene pairs, we count this as intra-TAD conservation. We performed the same analysis for gene pairs that lie in neighboring zebrafish TADs, from which we get an inter-TAD conservation score. Because the distances of intra-TAD gene pairs are lower than those of inter-TAD gene pairs, we divided the gene distances into three bins (Figure S1F, cumulative distribution of distances). We then plotted the observed intra-TAD conservation versus the inter-TAD conservation (see Figures 1C and S1E for a schematic representation). We found that the intra-TAD conservation is stronger than the inter-TAD score at every length scale. These results show that there is positive selection pressure within the vertebrate lineage to keep gene pairs in TADs together, implicating TADs as the mediator of selection in this process.

In mammals, loops (Rao et al., 2014) and TADs (Vietri Rudan et al., 2015) are demarcated by convergently oriented CTCF sites. We used ATAC-seq data (Gómez-Marín et al., 2015) derived from 24-hpf embryos to identify open chromatin regions (OCRs) containing a CTCF binding motif. We identified ∼37,000 OCRs with high-confidence CTCF motifs (STAR Methods). We plotted the orientation of the inferred CTCF binding sites relative to the TAD boundaries to show that CTCF binding sites are more numerous close to TAD boundaries (Figure 1D). When we stratify CTCF motifs based on their orientation, we find that close to the left/5′ boundary, the forward- or inward-pointing CTCF sites outnumber the reverse motifs (Figure 1D). At the right/3′ border, the opposite is found, showing the characteristic orientation seen in mammals. The interaction between convergently oriented CTCF sites located hundreds of kilobases apart can be explained by the loop extrusion model (Fudenberg et al., 2016, Sanborn et al., 2015), suggesting that loop extrusion may also be responsible for TAD formation in zebrafish.

Finally, mammalian genes within the same TAD tend to be temporally or spatially co-expressed (Symmons et al., 2014). To look into this in zebrafish, we used Tomo-seq data generated at the 15-somite stage to identify spatially co-expressed genes (Junker et al., 2014). We asked which neighboring genes at various distances were co-expressed. Upon stratifying co-expressed genes based on whether they lie in the same TAD, we found that neighboring genes that are co-expressed are more likely to be within the same TAD than the global average (Figure 1E; Figure S1G).

In summary, we show that the zebrafish genome is organized in TADs and that the TADs we observe have features similar to those of mammalian TADs.

### Zebrafish Chromosomes Lose TAD Structure during the m/z Transition

**Figure 2:**
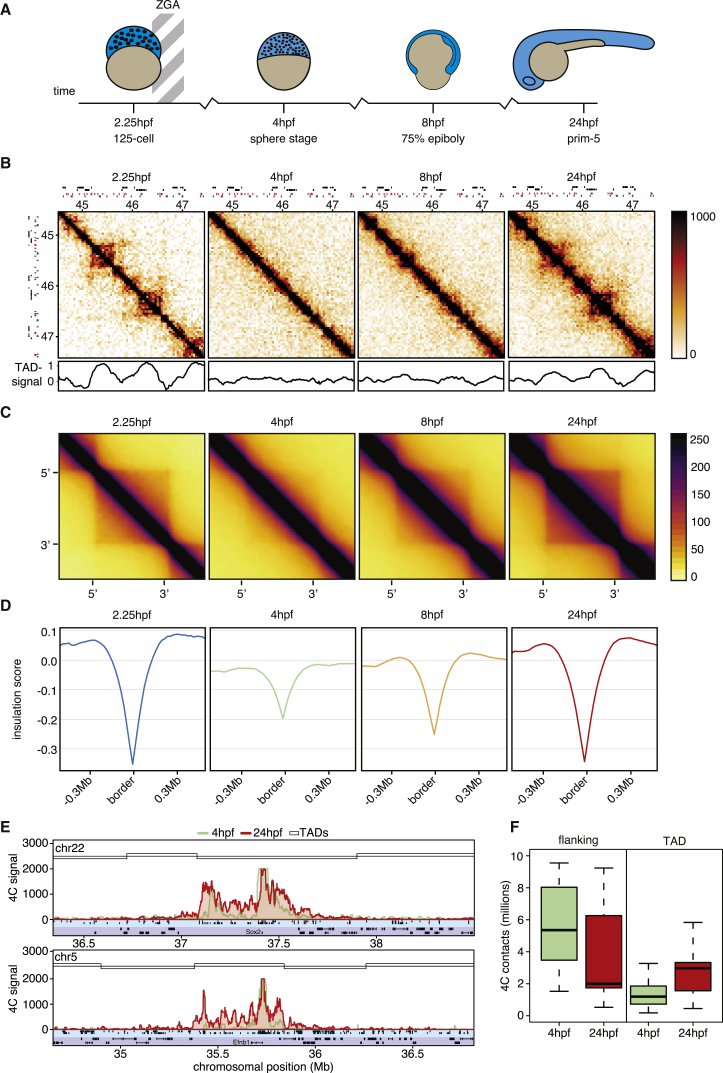
ZGA Is Accompanied by a Dramatic Loss of TAD Structure in Zebrafish (A) Schematic representation of the four developmental stages assayed by *in situ* Hi-C. (B) Zoom-in of a ∼4-Mb Hi-C contact matrix of chromosome 9 at 40-kb resolution, similar as Figure 1A. Below the plots, the TAD signal or insulation score is plotted. Insulation scores were calculated for Hi-C matrices with 20-kb resolution and a window size of 25 bins. (C) Aggregate TAD plots, based on TAD calls from 24 hpf, for all four Hi-C datasets. Hi-C data are the average of 2, 8, 9, and 4 biological replicates for 2.25-, 4-, 8-, and 24-hpf time points, respectively. (D) Insulation scores around 24-hpf TAD borders throughout zebrafish development, as indicated. (E) 4C-seq experiments show the contact frequency of the Sox2 TSS (upper panel) and an H3K27ac-enriched region (lower panel) at 4 hpf. The 24-hpf TADs are indicated in open rectangles. Below the 4C-seq plot, enhancers (light blue rectangle) and gene models (dark blue rectangle) are depicted. (F) Boxplot showing the quantification of the contact frequency in the 15-kb region flanking the viewpoint and the rest of the TAD measured in 11 4C-seq experiments at 4 and 24 hpf (p = 0.00054, paired Wilcoxon rank sum test, for flanking region comparison). Primers for the 4C viewpoints can be found in Table S1.

To study the dynamics of 3D genome organization throughout zebrafish development, we generated additional Hi-C maps at various developmental time points. Because we rely on clearly visible morphological structures, we chose 2.25 hpf (before ZGA), 4 hpf (post-ZGA), and 8 hpf (gastrulation) (Figure 2A). Visual inspection of the obtained contact matrices showed the organization of the zebrafish genome into TADs at 2.25 hpf (Figure 2B). However, after ZGA, there is a dramatic loss in TAD structure. At 8 hpf, TAD structures gradually reappear, leading to the TAD structures we see in 24-hpf embryos. To visualize the dynamics of TADs genome-wide, we generated plots showing the aggregate TAD signal (Figure 2C), showing that the loss of TAD structure at 4 hpf is a genome-wide phenomenon. To quantify TAD boundary strength in an alternative way, we also calculated the insulation score around TAD borders (Figure S2A). Aggregate plots of the insulation scores of 24-hpf TAD boundaries throughout zebrafish development show that the TAD boundary insulation is the weakest at 4 hpf and that this is the case for most TAD boundaries (Figure 2D; Figure S2B). Our Hi-C profiles are the sum of multiple independent template preparations from multiple independent collections of embryos. Analyses of the independent templates recapitulate our findings in the combined dataset (Figure S2C).

It is tempting to speculate that the loss of 3D genome organization is linked to the rapid rate of division of these cells, because previous work has shown that metaphase chromosomes show loss of TAD structure (Naumova et al., 2013). However, two lines of evidence lead us to be confident that this cannot be the full explanation. First, at 2.25 hpf, we see TAD structures, while at this time point, the rate of division is as high as, if not higher than, at 4 hpf. Second, image analysis of metaphase nuclei at the stages for which Hi-C maps were generated showed that most cells at 4 hpf are not in metaphase (Figures S2D–S2G).

To confirm the observations in the Hi-C data, we performed chromosome conformation capture coupled with sequencing (4C-seq) experiments and chose 4 and 24 hpf as the time points with the greatest difference. We designed viewpoints at putative enhancers, at TSSs, and close to TAD boundaries. We found that with the exception of the region flanking the viewpoint, the contact frequency within a TAD is lower at 4 hpf compared to 24 hpf (Figure 2E). When we systematically compare the contact frequency within the TAD (excluding the 15 kb flanking the viewpoint) between 4 and 24 hpf, we find that 11 of 11 viewpoints show an increase at 24 hpf (Figure 2F; Figure S3). However, some chromatin loops exist at 4 hpf, because we find that the TSS of Sox2 loops to a distal (>100 kb) cluster of enhancers (Figure 2E, upper panel).

What could be causing the loss of TADs at 4 hpf? Because TAD boundaries depend on CTCF in mouse embryonic stem cells (ESCs) (Nora et al., 2017), we tested whether the binding of CTCF was affected at 4 hpf. First, we analyzed an ATAC-seq dataset of 4-hpf embryos (Kaaij et al., 2016) and found almost 5-fold enrichment of CTCF motifs in the OCRs over a shifted control (14% of OCRs versus 2.8% of shifted OCRs), including the typical convergent orientation close to TAD borders (Figure S2H), suggesting that the relevant CTCF sites are accessible at 4 hpf. Second, we aligned a 4-hpf nucleosome positioning dataset (Zhang et al., 2014) on the 4- and 24-hpf CTCF-motif-containing OCRs and detected the characteristic nucleosome positioning pattern for the inferred CTCF binding sites (Figure S2I). These results imply that CTCF is bound to DNA and actively promoting nucleosome remodeling at 4 hpf. The observed lack of TAD structure at 4 hpf is likely not due to absence of CTCF.

In summary, in the period after the ZGA, the characteristic segmentation of interphase chromosomes into TADs is largely lost, even though certain chromatin loops can still be formed.

### Enrichment of Enhancer-Associated Histone Marks Negatively Correlates with TAD Boundary Strength

**Figure 3:**
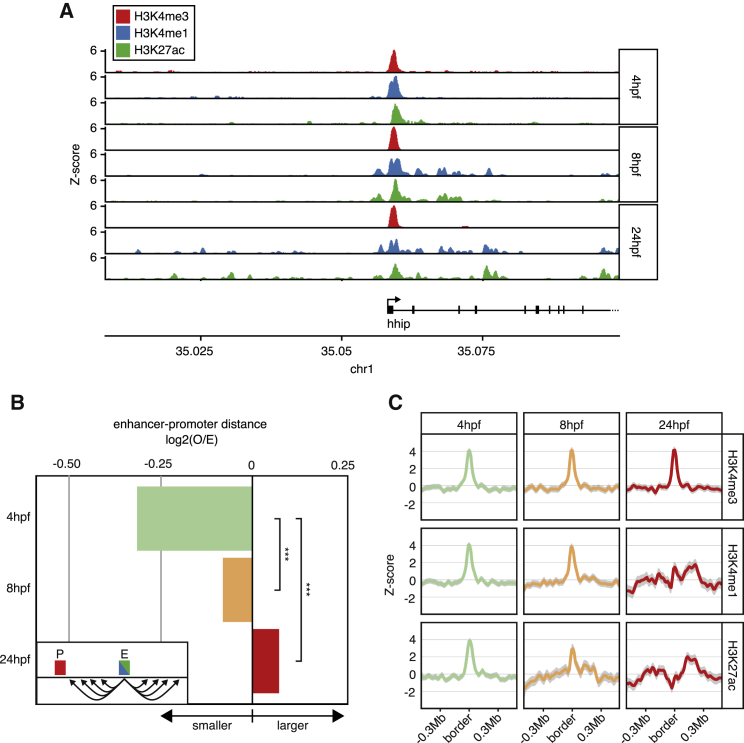
Dynamic Epigenomic Characteristics of TAD Boundaries throughout Development (A) ChIP-seq signal of H3K4me3, H3K4me1, and H3K27ac ChIP-seq datasets throughout zebrafish development at the *hhip* locus. (B) Barplot displaying the log2(O/E) (observed/expected) distance between TSS (H3K4me3+ regions) and the nearest active enhancer (defined as H3K4me1+/H3K27ac+ genomic regions) at three developmental time points. Expected values were calculated by local shuffling of the enhancers (STAR Methods). (C) *Z* score-normalized read densities over TAD borders of H3K4me3, H3K4me1, and H3K27ac ChIP-seq datasets, as indicated.

TADs are thought to act as regulatory scaffolds that facilitate long-range promoter-enhancer interactions (Symmons et al., 2014). We analyzed published chromatin immunoprecipitation sequencing (ChIP-seq) datasets (Bogdanovic et al., 2012) of the active promoter mark H3K4me3, poised enhancer mark H3K4me1, and active enhancer mark H3K27ac and found that distal enhancers increase during developmental progression for certain genes (Figure 3A). To determine whether this is a genome-wide effect, we calculated the distances between all active enhancers and the closest active TSS (Figure 3B). By aligning the 4-, 8-, and 24-hpf ChIP-seq data on the 24-hpf TAD boundaries, we investigated the distribution of these histone marks relative to the TAD boundaries throughout development (Figure 3C). We found that H3K4me3 was enriched around TAD boundaries, which is in agreement with the observation that mostly active genes are also enriched at TAD boundaries. Even at 4 hpf, when TAD boundaries are weaker, we see an enrichment of active promoter marks at boundaries. At this time point, we also see an enrichment of H3K4me1 and H3K27ac around TAD borders. Throughout development, however, this enrichment is gradually lost. Our observations are consistent with a model in which distal regulatory elements cannot regulate genes over long distances in the absence of TADs and are therefore selected against.

### Chromosome Compartmentalization Is Lost and Subsequently Established throughout Development

**Figure 4:**
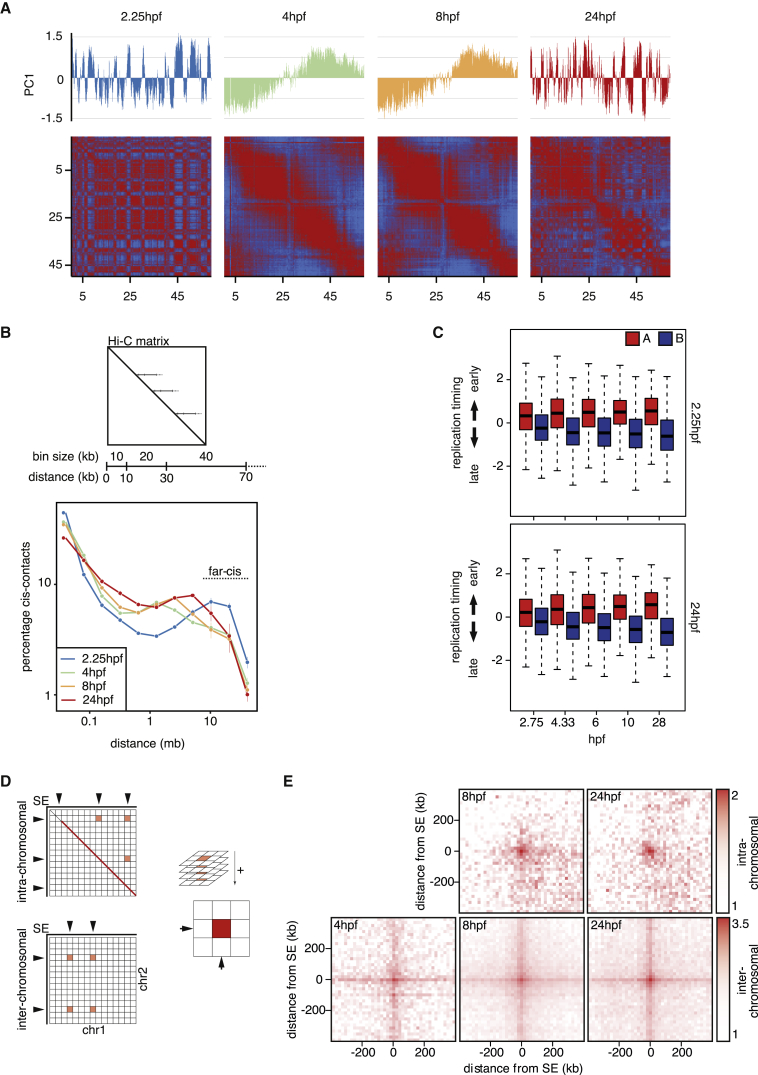
A and B Compartments Are Lost after ZGA and Slowly Re-established throughout Development (A) HOMER-derived PC1 values of chromosome 1 at the indicated time points (upper panels). The lower panels display correlation matrices obtained at 500-kb resolution of chromosome 1 (red = 1 and blue = −1). (B) Relative contact frequency plot showing the percentage of contacts as a function of distance; bin sizes increase exponentially. The upper panel shows a schematic explanation of the calculation of the number of contacts (contact frequency) for every position in the genome with other regions on the same chromosome. Because contact frequency decreases with distance, we use exponentially increasing bin sizes. (C) Boxplots showing replication time for genomic regions called as A (red) and B (blue) compartments at 2.25 hpf (upper boxplot) and 24 hpf (lower boxplot). (D) Schematic explanation of the PE-SCAn method. The average contact frequency is calculated for all pairwise super-enhancer combinations (see STAR Methods for a detailed explanation). (E) Top row shows PE-SCAn results of intra-chromosomal interactions between super-enhancers called at 8 and 24 hpf in the respective time points. Bottom row shows average pairwise contact frequency between super-enhancers on different chromosomes.

When we inspect our Hi-C maps of the various time points, we find dramatic differences throughout development in chromosome compartmentalization. Compartmentalization is strong at 2.25 hpf (Figure 4A). The 2.25-hpf time point is before ZGA, which means there is no transcription occurring, showing that chromosome compartmentalization can take place without transcription, in line with our previous observation that the inactive X chromosome adopts the organization of the active X chromosome after the knockout of *Xist* without gene activation (Splinter et al., 2011). When we look at the 4-hpf embryo genome, we see that ZGA is accompanied by a near-complete loss of compartmentalization (Figure 4A). Similar to our observations for TAD organization, we see that compartmentalization increases from 8 hpf onward. The loss and gain in compartmentalization are found in multiple independent templates (Figures S4A and S4B). Next, we analyzed three aspects of genome biology in relation to these observations: long-range intra-chromosomal contacts, replication timing, and clustering of super-enhancers.

We calculated how intra-chromosomal contacts are distributed as a function of their distance. To this end, we bin the contacts based on their distance. We observe that the two time points with clear A/B compartmentalization, 2.25 and 24 hpf, have the highest relative contact frequency between genomic regions that are >5 Mb apart (Figure 4B; Figure S4C).

One of the features that has been shown to be most strongly correlated with A/B compartmentalization is replication timing. A compartments generally replicate early in S phase, whereas B compartments are late replicating (Ryba et al., 2010). To determine whether a similar correlation exists in zebrafish, we used a recently published dataset that measured replication timing throughout zebrafish development at roughly the same time points for which we have generated Hi-C maps (Siefert et al., 2017). We determined the distribution of replication timing at 28 hpf in 24-hpf A and B compartments and found a strong association (Figure 4C). Also at 4.33 hpf, when ostensibly there are no TADs and compartments, the replication timing data show a clear association with the compartments at 2.25 and 24 hpf, suggesting that compartments and replication timing can be uncoupled. This is supported by observations of the 2.25-hpf compartments. Although the A/B compartments at 2.25 hpf show an association with replication timing at 2.75 hpf, the A/B compartmentalization at 2.25 hpf is more predictive of replication timing at 28 hpf. This shows that replication timing domains can form in the absence of compartments and suggests that other, perhaps DNA sequence-intrinsic characteristics dictate replication timing. Therefore, even though there is a clear correlation between A/B compartments and replication timing, the relationship is likely more complicated than one dictating the other.

We (Krijger et al., 2016, de Wit et al., 2013) and others (Beagrie et al., 2017, Rao et al., 2017) have shown that super-enhancers show preferred interactions in the genome over large distances (>10 Mb). To determine whether super-enhancers showed clustering in the 3D genome of the developing embryo, we used paired-end spatial chromatin analysis (PE-SCAn) to perform pairwise alignment of the Hi-C data on all intra- and inter-chromosomal super-enhancer combinations (STAR Methods; Figure 4D). At 4 hpf, we could not call enough super-enhancers to perform PE-SCAn for intra-chromosomal interactions. At 8 and 24 hpf, we see clear enrichment of spatial interactions for super-enhancer combinations (Figure 4E). These observations are replicated for inter-chromosomal interactions (Figure 4E). This is particularly notable given that at 4.33 and 8 hpf, there is only weak A/B compartmentalization and TAD formation, showing that super-enhancer clusters can form independently of both TADs and A/B compartments.

## Discussion

We show here that the 3D organization of the genome in the developing zebrafish embryo shows three clear stages. Strong compartmentalization and TAD-like structures are apparent directly after fertilization (stage 1). After ZGA, these structures are lost (stage 2). Finally, at 24 hpf, both compartments and TADs are re-established (stage 3). Although TADs and A/B compartments are strongly associated with transcription, we show here that TADs and compartments can form in the absence of transcription, indicating once more that transcription is not a prerequisite for compartmentalization. Conversely, we also show that expression does not require TADs and compartments per se.

When we compare the developmental dynamics of the 3D genome in zebrafish embryos with *Drosophila* or mouse, what stands out is the organized chromosomes at the earliest assayed time point (stage 1). In mouse, oocytes and female pronuclei lack compartments, whereas sperm and male pronuclei show compartmentalization (Flyamer et al., 2017, Ke et al., 2017). In the zygote and 2-cell stages, 3D genome features such as compartments and TADs are not present. Upon further development (i.e., 4-cell and 8-cell stages), TADs emerge, independent of transcription. Note the different timescales involved here: whereas in zebrafish the dynamics of the 3D genome occurred within the first 24 hpf, in mouse no cell division occurred in this time frame. In *Drosophila*, however, development was quicker, reaching the 10^th^ nuclear cycle (i.e., 512 cells) 2 hours after fertilization (Gilbert, 2000). At nuclear cycle 12, after the minor ZGA, there is a clear absence of chromatin architecture (Hug et al., 2017). The embryos at the 2.25-hpf time point that we assay in our study have undergone 7 cell divisions and are still transcriptionally silent. It will be interesting to see whether, at earlier developmental time points in *Drosophila* embryos, the 3D architectural features are absent, as in mouse, or they have organized chromatin architecture, similar to zebrafish embryos.

The formation of TADs depends on the binding of Cohesin to DNA. In interphase nuclei, loss of Cohesin or loss of factors that load Cohesin on the DNA results in a strongly diminished TAD organization; however, this is accompanied by an increase in compartmentalization (Haarhuis et al., 2017, Rao et al., 2017, Schwarzer et al., 2017). Stabilization of Cohesin on DNA can result in strongly diminished compartmentalization but results in the formation of longer CTCF/Cohesin loops (Gassler et al., 2017, Haarhuis et al., 2017, Wutz et al., 2017). The lack of TADs and compartments is most reminiscent of metaphase chromosomes, in which both compartments and TADs have disappeared because of the activity of the Condensin I and II complexes (Gibcus et al., 2018). However, in our microscopy analysis, only a minority of chromosomes show the characteristic rod-shaped chromosomes of metaphase. A possible explanation is that full decondensation is prevented in cells that are in stage 2. This could be achieved if the Condensin complexes remain active throughout interphase. Even though the exact role of Condensin in interphase chromosome organization is not clear, details of it are starting to emerge (Hirano, 2016).

Alternatively, decreased activity of the Cohesin complex could be an explanation for the loss of TADs; however, this would require an inhibitor for the formation of compartments (described earlier). It has been suggested that compartments are phase-separated domains whose formation is countered by loop extrusion (Rao et al., 2017, Schwarzer et al., 2017). Heterochromatin protein 1 (HP1) has been suggested to play a role in phase separation of heterochromatin domains (Larson et al., 2017, Strom et al., 2017), but other factors are also likely involved. Proteins or post-translational histone modifications that counter phase separation may decrease compartmentalization. For example, phosphorylation of the 10^th^ serine and acetylation of the 14^th^ lysine of histone H3 interferes with the binding of HP1 (Mateescu et al., 2004) and may thereby counter compartmentalization.

An open question remains whether the changes we observe are gradual (occurring over multiple nuclear cycles) or abrupt (occurring from one nuclear cycle to the next) and at which developmental time point they occur. Using exciting technologies such as single-cell Hi-C (Nagano et al., 2013), it should be possible to temporally resolve the observed transitions.

We believe that the systemic re-programming of the 3D genome in the developing zebrafish embryo is a promising model to study fundamental questions in nuclear organization.

## STAR★Methods

### Key Resources Table

**Table:** 

REAGENT or RESOURCE	SOURCE	IDENTIFIER
**Chemicals, Peptides, and Recombinant Proteins**		

T4 DNA Ligase Buffer Pack	Promega	C1263
T4 DNA Ligase	Sigma	10799009001
TrypLE Express Enzyme	Life	12605-010
*Genomic DNA Reagents*	Agilent Technolgies	5067-5366
T4 DNA Ligase Buffer Pack	Promega	C1263
*Genomic DNA Screen Tape*	Agilent Technolgies	5067-5365
cOmplete, Mini, EDTA-free	Roche	11836170001
Csp6I	Thermo	ER0211
DPnII	New England Biolabs	R0543M
NEBNext High-Fidelity 2X PCR Master Mix	NEB	M0541L
Phenol:Chloroform:IAA	Life	AM9730
Sodium Dodecyl Sulfate (SDS), 20% Solution	piercenet	62202
100uM cell strainer	Sigma	CLS431752-50EA

**Experimental Models: Organisms/Strains**		

Zebrafish (TU and TLF strain)	N/A	N/A

**Oligonucleotides**		

4C-Seq primers	Table S1, this study	N/A

**Software and Algorithms**		

HiC-pro v2.9	Servant et al., 2015	https://github.com/nservant/HiC-Pro
Bowtie v2.3.3.1	Langmead and Salzberg, 2012	http://bowtie-bio.sourceforge.net/bowtie2/index.shtml
MACS v2.1	Zhang et al., 2008	https://github.com/taoliu/MACS
FIMO v4.12	Grant et al., 2011	http://meme-suite.org/doc/fimo.html
HiCseg v1.1	Lévy-Leduc et al., 2014	https://cran.r-project.org/web/packages/HiCseg/index.html
CaTCH	Zhan et al., 2017	https://github.com/zhanyinx/CaTCH_R
deeptools v2.5	Ramírez et al., 2016	https://github.com/fidelram/deepTools
HOMER v4.9	Heinz et al., 2010	http://homer.ucsd.edu/homer/index.html

### Contact for Reagent and Resource Sharing

Further information and requests for resources and reagents should be directed to and will be fulfilled by the Lead Contact, Dr. Elzo de Wit (e.d.wit@nki.nl).

### Experimental Model and Subject Details

Zebrafish (TU and TLF strain) were kept under standard conditions (Westerfield, 2000) and staged according to (Kimmel et al., 1995). To obtain large quantities of embryos with approximately the same developmental stage fish were mated for only 10-15 min. Every batch of embryos was staged based on morphological features; we made sure that the vast majority of embryos were at the correct developmental stage. The developmental stages were picked based on the presence of clear morphological features. In the case of the 2.25hpf and 4hpf time points we wanted an embryo population that was either pre-ZGA or post-ZGA and not a mixture of both. Animals were housed at the Institute of Molecular Biology in Mainz under licenses of the local government and in accordance with German bioethical regulations.

### Methods Details

#### *In situ* Hi-C

Carefully staged embryos were dechorionated, deyolked and made single cell in three consecutive steps. First embryos were dounced and spun down at 500 r*cf.* at 4 degrees. The precipitate was thereafter incubated in 2 mL of TRIPLE (life, Cat# 12605-010) for 5 min at RT after which 10% end concentration FBS was added. This solution was filtered using a 100uM cell strainer (Sigma, Cat# CLS431752-50EA) and cells were spun down at 500 r*cf.* at 4 degrees to collect the single cells. These cells were subsequently processed following the standard 4C protocol using DpnII as the restriction enzyme. Successful digestion and ligation was confirmed using the agilent TAPEStation. We omitted the usual biotin incorporation and enrichment step due to the low amounts of DNA obtained from the early developmental stages. Reverse cross-linked DNA was quantified using QUBIT (thermo fisher) and subsequently sheared to 700-900bp using the Covaris. We subsequently generated paired-end deep-sequencing libraries using the Ovation Ultralow Library Prep kit (Nugen). Libraries were sequenced on the HiSeq or NextSeq.

Raw sequence data were mapped and processed to the GRCz10 reference genome using HiC-Pro v2.9 (Servant et al., 2015). Hi-C data are available from GEO accession GSE105013.

#### 4C-seq

4C-Seq was performed as described previously and above under the Hi-C section (Kaaij et al., 2016, van de Werken et al., 2012). Briefly, after obtaining a single cell suspension, crosslinking of the nuclei, primary digestion with DpnII and ligation, the DNA was reverse crosslinked o/n at 65°C. DNA was isolated by phenol/chloroform extraction and subsequently digested with a 2^nd^ restriction enzyme (Csp6I). To create circular DNA molecules, digested DNA was ligated under diluted conditions (10ml). DNA was precipitated with 1/10 volume 3M sodium acetate and 1 volume isopropanol. DNA was quantified using QUBIT (thermo fisher). 4C PCR was done using NEB-Next High-Fidelity (NEB,M0541) in 4 separate PCR reactions using ∼1-200ng per PCR. See Table S1 for primer sequences. 4C-seq data are available from GEO accession GSE105014.

#### Cell cycle quantification

Carefully staged embryos were manually dechorionated and subsequently incubated o/n with 1ug/ml DAPI (Roche) in PBST. Stained embryos were washed three times with PBST. Embryos were imaged using an Upright Spinning disk Confocal Microscope (Zeiss). Quantification of cell cycle stages was done manually.

#### ATAC-seq

ATAC-seq data were taken from (Gómez-Marín et al., 2015, Kaaij et al., 2016). The raw sequencing data were mapped using bowtie2 using the GRCz10 reference genome with default parameters. We called peaks using MACS2 (Zhang et al., 2008) with parameters -g 1.5e9,–nomodel,–shift −100 and–extsize 200. To identify CTCF-motifs within the ATAC-seq peaks, we used FIMO (Grant et al., 2011) of the MEME suite. For this, we searched for the vertebrate CTCF-motif (Jaspar ID: MA0139.1)(Mathelier et al., 2016).

#### TAD-analysis

TADs were called with HiCseq (Lévy-Leduc et al., 2014), using the 20kb matrices of 24hpf. Next, we counted the number of CTCF-motifs in forward or reverse orientation in ten 10kb bins from the TAD-border. Since this resulted in a clear enrichment of CTCF on the TAD-border, consistent with observations in other animals, we opted to use this information for CaTCH (Zhan et al., 2017). With CaTCH, we were able to call TADs, with the *a priori* information about the enrichment of CTCF on boundaries. Unfortunately, due to scaffolding errors and uncovered regions in the reference genome there were erroneous TAD calls. The reason for this is that scaffolding errors and regions without coverage resemble (strong) TAD borders. We therefore first performed an automatic filtering of TAD borders by removing TAD borders that overlap with Hi-C bins that had no coverage. Second, because scaffolding errors are clear in the Hi-C matrix (examples can be seen in (Dudchenko et al., 2017)), but difficult to detect automatically, we performed further manual curation of our set of TAD borders.

Insulation-scores were calculated as described in (Crane et al., 2015), using a window-size of 500kb. The generated tracks were aligned on the 24hpf 5′ TAD-borders using deeptools2 (Ramírez et al., 2016) with the following parameters: -a 500kb, -b 500kb –bs 10kb.

The Aggregate TAD Analysis was performed as in (Haarhuis et al., 2017) using the 24hpf CatTCH TADs and the 20kb matrices of each time-point. In short, this method takes every TAD and its surrounding region and resizes them to a 100x100 matrix. These resized matrices are then averaged across and plotted using ggplot2.

#### Conservation-analysis

To quantify the conservation of TADs in zebrafish, we look whether two orthologous genes within one zebrafish-TAD are within 1Mb of each other in another species. To measure the conservation between TADs, we asked whether two orthologous genes in two neighboring zebrafish TADs are within 1Mb of each other. We only use genes that have strict orthologs (i.e., a zebrafish gene can only have a single ortholog in a comparison species). We then quantify both these queries by using the percentage of TADs with at least one gene-pair within 1Mb. The gene-builds used were from EnsEMBL genes 90 and queried using Biomart. Because distance-distributions of intra- and inter-TAD gene pairs are not similar (Figure S1F), we computed the conservation scores for three distance-bins. Because the number of gene pairs differ per distance bin between intra- and inter-TAD we subsample the largest group to the smallest group. For a proper representation we randomly subsample 100 times.

#### ChIP-seq data

ChIP-seq data were downloaded from GEO (GSE32483) and mapped with Bowtie version 2.3.3 (Langmead and Salzberg, 2012) using default parameters. BigWig- and BED-tracks were generated with MACS2, (Zhang et al., 2008) using the –bdg to generate pileups. The alignment of histone marks on TAD-borders was done by aligning the bigwig-tracks on the 5′ border with deeptools2 (Ramírez et al., 2016). Super-enhancers were called using the HOMER version 4.9 (Heinz et al., 2010), taking the H3K27ac mark as input-data. To calculate the super-enhancer spatial interactions, we used PE-SCAn (de Wit et al., 2013); for intra-chromosomal interactions we only use interaction that are >5Mb apart (“far-cis”).

To quantify the distance between enhancer and promoters, we defined both promoters and enhancers in every time point. We define a promoter as a region with an H3K4me3 peak that is within 2kb of a transcriptional start site (TSS), taken from EnsEMBL gene annotation release 90. An enhancer is defined as a region with both H3K27ac and H3K4me1 peaks, and no overlap with an H3K4me3 peak. Next, we found the closest enhancer for every promoter and calculated the distance between these pairs. Finally, we determined the observed over expected ratio by randomly shuffling the positions of the enhancers. We shuffled the positions within a 1Mb window around the enhancer. The average observed value was divided by the average randomized value for every time point.

#### Compartment-analysis

Homer was used to perform a 100kb resolution principal component analysis for each time-point, using the H3K4me1 ChIP-seq data as annotation of active regions. To find the most informative principal component, we searched for the best correlation between a PC and GC-content as proposed in (Naumova et al., 2013).

#### PE-SCAn

For all pairwise combinations of super-enhancers along a chromosome a submatrix is extracted from the Hi-C matrix. The average of all these submatrices is calculated to determine the signal. Next, the super enhancer positions are shifted by 1Mb and the same procedure is repeated to generate an average random matrix (not shown). The average real matrix is normalized by the median of average random matrix to determine whether super-enhancers preferentially interact. Note that super-enhancer pairs that lie within 5Mb of each other are not taken along in this analysis (indicated by red diagonal). For interchromosomal super enhancer comparisons the same procedure is followed.

#### Co-expression

Tomo-seq data were taken from (Junker et al., 2014) and GSE59873. In order to determine whether genes in the same TAD have a higher probability to be co-expressed, we calculated the Spearman rank-correlation for all neighboring genes on a chromosome. We selected gene pairs that had a correlation coefficient above a certain threshold (ρ > 0.4, ρ > 0.5 or ρ > 0.6). We stratified the gene pairs whether they were found in the same TAD or not. We scored the correlation coefficients at different distances, where d = 0 represents directly neighboring genes, d = 1 are gene pairs with one gene in between, et cetera. We performed our analysis separately on three biological Tomo-seq replicates. As a quality filter, we removed Tomo-seq sections in which less than 6000 genes were detected.

#### Replication timing

Replication timing data were taken from (Siefert et al., 2017) and GSE85713. Biological replicates were averaged per time point. The replication timing scores were stratified based on whether a region was in the A compartment and in the B compartment.

#### Nucleosome positioning

Nucleosome positioning data were taken from (Zhang et al., 2014) and GSE44269. Single-end reads were mapped to the GRCz10 reference genome and intersected with 4hpf and 24hpf inferred CTCF binding sites (see above) using bedtools window (Quinlan and Hall, 2010) with a window size of 1kb. Nucleosome center positions were inferred by adding 73 (i.e., 147/2) to reads mapped to the plus strand and subtracting 24 from reads mapped to the minus strand (i.e., 147/2 – 49, where 49 is the length of the read). Only reads with a mapping quality > 10 and CTCF motifs with a FIMO score > 12 were taken along in the alignment analysis.

#### Housekeeping-genes

Shannon-Entropy as a measure for tissue-specificity has been originally introduced by (Schug et al., 2005). Please note that Schug et al. use an additional statistical approach to define whether the gene is specific to a particular sample and we do not do that in our analysis. A detailed description of the benefits of using this approach over other approaches is provided by Schug *et* al. In brief, conventional strategies often define tissue specificity as the relative expression in a sample compared to all samples considered. In contrast Shannon-Entropy measurements take into account the observed expression levels in all samples and when measuring the specificity of expression of gene A in one sample it takes into account the distribution of expression levels of gene A in all samples. To compute the Shannon-Entropy we used the following formula where P contains the TPM values for a given gene across *n* RNA-seq experiments. First the expression values are normalized

$$P_{i,norm}=P_{i}/\sum_{i=1}^{n}P_{i}$$

Next the Shannon entropy is calculated as follows:

$$H\left(P\right)=-\sum_{i=1}^{n}P_{i,norm}\;\log_{2}\;P_{i,norm}$$

A high Shannon entropy score indicates tissue-specificity, whereas a low Shannon entropy score indicates the genes is more broadly expressed. The top and bottom 1000 genes are classified as tissue-specific and housekeeping, respectively, and were used for further analysis.

TPM values were obtained using RNA-seq data from the following SRA files SRR1821783; SRR1821784; SRR1821807; SRR1821808; SRR1821827; SRR1821828; SRR2959456; SRR1616928; SRR1616929; SRR1914392; SRR957180; SRR1205160; SRR1205161; SRR372787; SRR372788; SRR372789; SRR372790; SRR372791; SRR372792; SRR372793; SRR372794; SRR372795; SRR372796; SRR372797; SRR372798; SRR372799; SRR372800; SRR372801; SRR372802; SRR372803 (Friedmann et al., 2015, Jiang et al., 2014, Kaufman et al., 2016, Pauli et al., 2012, Wang et al., 2015).

### Quantification and Statistical Analyses

All p values were calculated in R and interpreted as indicated in the text.

### Data and Software Availability

The sequencing data generated in this study have been deposited in the NCBI Gene Expression Omnibus (GEO) repository under the accession number GEO: GSE105013 and GSE105014.

## Supplemental Information

Document S1. Figures S1–S4 and Table S1

Document S2. Article plus Supplemental Information
