# Supplementary material for: Systemic Loss and Gain of Chromatin Architecture throughout Zebrafish Development
Source: Cell Rep. 2018 Jul 3;24(1):1–10.e4. doi: 10.1016/j.celrep.2018.06.003 (PMC6047509; doi:10.1016/j.celrep.2018.06.003)
Supplement: Document S1. Figures S1–S4 and Table S1 [file mmc1.pdf]

Cell Reports, Volume *24*

Supplemental Information

Systemic Loss and Gain of Chromatin Architecture throughout Zebrafish Development

Lucas J.T. Kaaij, Robin H. van der Weide, René F. Ketting, and Elzo de Wit

Table S1

view 1	view 1	primer sequences
>chr24:3031279-3031965_fwd	24:3031279-3031965_fwd_BC1	AATGATACGGCGACCACCGAGATCTACACTCTTTCCCTACACGACGCTCTTCCGATCTACACGTCGCTTATATCGGGATC
ACGTCGCTTATATCGGGATC	24:3031279-3031965_fwd_BC2	AATGATACGGCGACCACCGAGATCTACACTCTTTCCCTACACGACGCTCTTCCGATCTtgACGTCGCTTATATCGGGATC
>chr24:3031279-3031965_rev	24:3031279-3031965_rev	CAAGCAGAAGACGGCATACGAGATCGGTCTCGGCATTCCTGCTGAACCGCTCTTCCGATCTGATGCCCTACACTCTCATTC
GATGCCCTACACTCTCATTC		
view 2	view 2	
>chr3:18723607-18724650_fwd	3:18723607-18724650_fwd_BC1	AATGATACGGCGACCACCGAGATCTACACTCTTTCCCTACACGACGCTCTTCCGATCTACCTTCCATATACATGCAGATC
CTTCCATATACATGCAGATC	3:18723607-18724650_fwd_BC2	AATGATACGGCGACCACCGAGATCTACACTCTTTCCCTACACGACGCTCTTCCGATCTtgCTTCCATATACATGCAGATC
>chr3:18723607-18724650_rev	3:18723607-18724650_rev	CAAGCAGAAGACGGCATACGAGATCGGTCTCGGCATTCCTGCTGAACCGCTCTTCCGATCTAAAATTTTCATATTGGGGTG
AAAATTTTCATATTGGGGTG		
view3	view3	
>chr4:11210227-11210713_fwd	4:11210227-11210713_fwd_BC1	AATGATACGGCGACCACCGAGATCTACACTCTTTCCCTACACGACGCTCTTCCGATCTACATGAATGAATCTGACTGATC
ATGAATGAATCTGACTGATC	4:11210227-11210713_fwd_BC2	AATGATACGGCGACCACCGAGATCTACACTCTTTCCCTACACGACGCTCTTCCGATCTtgATGAATGAATCTGACTGATC
>chr4:11210227-11210713_rev	4:11210227-11210713_rev	CAAGCAGAAGACGGCATACGAGATCGGTCTCGGCATTCCTGCTGAACCGCTCTTCCGATCTTGAAACAAGGATTATTCCTC[illegible]
TGAAACAAGGATTATTCCTCA		
view4	view4	
>chr10:21909251-21909539_fwd	10:21909251-21909539_fwd_BC1	AATGATACGGCGACCACCGAGATCTACACTCTTTCCCTACACGACGCTCTTCCGATCTACAGAAACAAAAAGTTTAGATC
AGAAACAAAAAGTTTAGATC	10:21909251-21909539_fwd_BC2	AATGATACGGCGACCACCGAGATCTACACTCTTTCCCTACACGACGCTCTTCCGATCTtgAGAAACAAAAAGTTTAGATC
>chr10:21909251-21909539_rev	10:21909251-21909539_rev	CAAGCAGAAGACGGCATACGAGATCGGTCTCGGCATTCCTGCTGAACCGCTCTTCCGATCTACAGAGCCATTTATGCAGAC
ACAGAGCCATTTATGCAGAC		
view5 (check distance from viewpoint)	view5 (check distance from viewpoint)	
>chr22:37421871-37422581_fwd	22:37421871-37422581_fwd_BC1	AATGATACGGCGACCACCGAGATCTACACTCTTTCCCTACACGACGCTCTTCCGATCTACGCGACTGGACGCTATGGATC
GCGACTGGACGCTATGGATC	22:37421871-37422581_fwd_BC2	AATGATACGGCGACCACCGAGATCTACACTCTTTCCCTACACGACGCTCTTCCGATCTtgGCGACTGGACGCTATGGATC
>chr22:37421871-37422581_rev	22:37421871-37422581_rev	CAAGCAGAAGACGGCATACGAGATCGGTCTCGGCATTCCTGCTGAACCGCTCTTCCGATCTTTCTGCAATCCAAATTAGCT
TTCTGCAATCCAAATTAGCT		
view 6	view 6	
>chr24:12689902-12691922_fwd	24:12689902-12691922_fwd_BC1	AATGATACGGCGACCACCGAGATCTACACTCTTTCCCTACACGACGCTCTTCCGATCTACAGGCCTAATGAATGTAGATC
AGGCCTAATGAATGTAGATC	24:12689902-12691922_fwd_BC2	AATGATACGGCGACCACCGAGATCTACACTCTTTCCCTACACGACGCTCTTCCGATCTtgAGGCCTAATGAATGTAGATC
>chr24:12689902-12691922_rev	24:12689902-12691922_rev	CAAGCAGAAGACGGCATACGAGATCGGTCTCGGCATTCCTGCTGAACCGCTCTTCCGATCTCTTCAATCAGCATCCGTTTT
CTTCAATCAGCATCCGTTTT		
view7	view7	
>chr21:13593987-13594356_fwd	21:13593987-13594356_fwd_BC1	AATGATACGGCGACCACCGAGATCTACACTCTTTCCCTACACGACGCTCTTCCGATCTACAACCTGTGTGGGCCAGGATC
AACCTGTGTGGGCCAGGATC	21:13593987-13594356_fwd_BC2	AATGATACGGCGACCACCGAGATCTACACTCTTTCCCTACACGACGCTCTTCCGATCTtgAACCTGTGTGGGCCAGGATC
>chr21:13593987-13594356_rev	21:13593987-13594356_rev	CAAGCAGAAGACGGCATACGAGATCGGTCTCGGCATTCCTGCTGAACCGCTCTTCCGATCTCTGCCCTCTTATGCATATTT
CTGCCCTCTTATGCATATTT		

view8	view8	
>chr5:35734021-35734441_fwd	5:35734021-35734441_fwd_BC1	AATGATACGGCGACCACCGAGATCTACACTCTTTCCCTACACGACGCTCTTCCGATCTACTATCAGCAGCAGAAAAGATC
TATCAGCAGCAGAAAAGATC	5:35734021-35734441_fwd_BC2	AATGATACGGCGACCACCGAGATCTACACTCTTTCCCTACACGACGCTCTTCCGATCTtgTATCAGCAGCAGAAAAGATC
>chr5:35734021-35734441_rev	5:35734021-35734441_rev	CAAGCAGAAGACGGCATACGAGATCGGTCTCGGCATTCCTGCTGAACCGCTCTTCCGATCTAGGATTCCAAGGGTTTACAT
AGGATTCCAAGGGTTTACAT		
view9	view9	
>chr12:34629626-34629939_fwd	12:34629626-34629939_fwd_BC1	AATGATACGGCGACCACCGAGATCTACACTCTTTCCCTACACGACGCTCTTCCGATCTACCATTCTTTAATCTTCAGATC
CATTCTTTAATCTTCAGATC	12:34629626-34629939_fwd_BC2	AATGATACGGCGACCACCGAGATCTACACTCTTTCCCTACACGACGCTCTTCCGATCTtgCATTCTTTAATCTTCAGATC
>chr12:34629626-34629939_rev	12:34629626-34629939_rev	CAAGCAGAAGACGGCATACGAGATCGGTCTCGGCATTCCTGCTGAACCGCTCTTCCGATCTGCTCCATCATGTATTCGATT
GCTCCATCATGTATTCGATT		
view10	view10	
>chr22:18522392-18522640_fwd	22:18522392-18522640_fwd_BC1	AATGATACGGCGACCACCGAGATCTACACTCTTTCCCTACACGACGCTCTTCCGATCTACATAACCGTAACAAAGGGATC
ATAACCGTAACAAAGGGATC	22:18522392-18522640_fwd_BC2	AATGATACGGCGACCACCGAGATCTACACTCTTTCCCTACACGACGCTCTTCCGATCTtgATAACCGTAACAAAGGGATC
>chr22:18522392-18522640_rev	22:18522392-18522640_rev	CAAGCAGAAGACGGCATACGAGATCGGTCTCGGCATTCCTGCTGAACCGCTCTTCCGATCTCCAATGAAATAGTGGAGAGC
CCAATGAAATAGTGGAGAGC		
view11 (check distance to original view	view11 (check distance to original viewpoint)	
>chr6:32114060-32114600_fwd	6:32114060-32114600_fwd_BC1	AATGATACGGCGACCACCGAGATCTACACTCTTTCCCTACACGACGCTCTTCCGATCTACTGTTAAAAATGAGCTGGATC
TGTTAAAAATGAGCTGGATC	6:32114060-32114600_fwd_BC2	AATGATACGGCGACCACCGAGATCTACACTCTTTCCCTACACGACGCTCTTCCGATCTtgTGTTAAAAATGAGCTGGATC
>chr6:32114060-32114600_rev	6:32114060-32114600_rev	CAAGCAGAAGACGGCATACGAGATCGGTCTCGGCATTCCTGCTGAACCGCTCTTCCGATCTTGAAGATTGCCATTTTCTCT
TGAAGATTGCCATTTTCTCT		
view12	view12	
>chr3:28876830-28877096_fwd	3:28876830-28877096_fwd_BC1	AATGATACGGCGACCACCGAGATCTACACTCTTTCCCTACACGACGCTCTTCCGATCTACATATTCAGTCTCCCAAGATC
ATATTCAGTCTCCCAAGATC	3:28876830-28877096_fwd_BC2	AATGATACGGCGACCACCGAGATCTACACTCTTTCCCTACACGACGCTCTTCCGATCTtgATATTCAGTCTCCCAAGATC
>chr3:28876830-28877096_rev	3:28876830-28877096_rev_BC2	CAAGCAGAAGACGGCATACGAGATCGGTCTCGGCATTCCTGCTGAACCGCTCTTCCGATCTGGAGAGTAAATTATGGCACG
GGAGAGTAAATTATGGCACG		

Table S1 Primer sequences used for 4C-seq. Related to STAR Methods

Supplemental figure 1

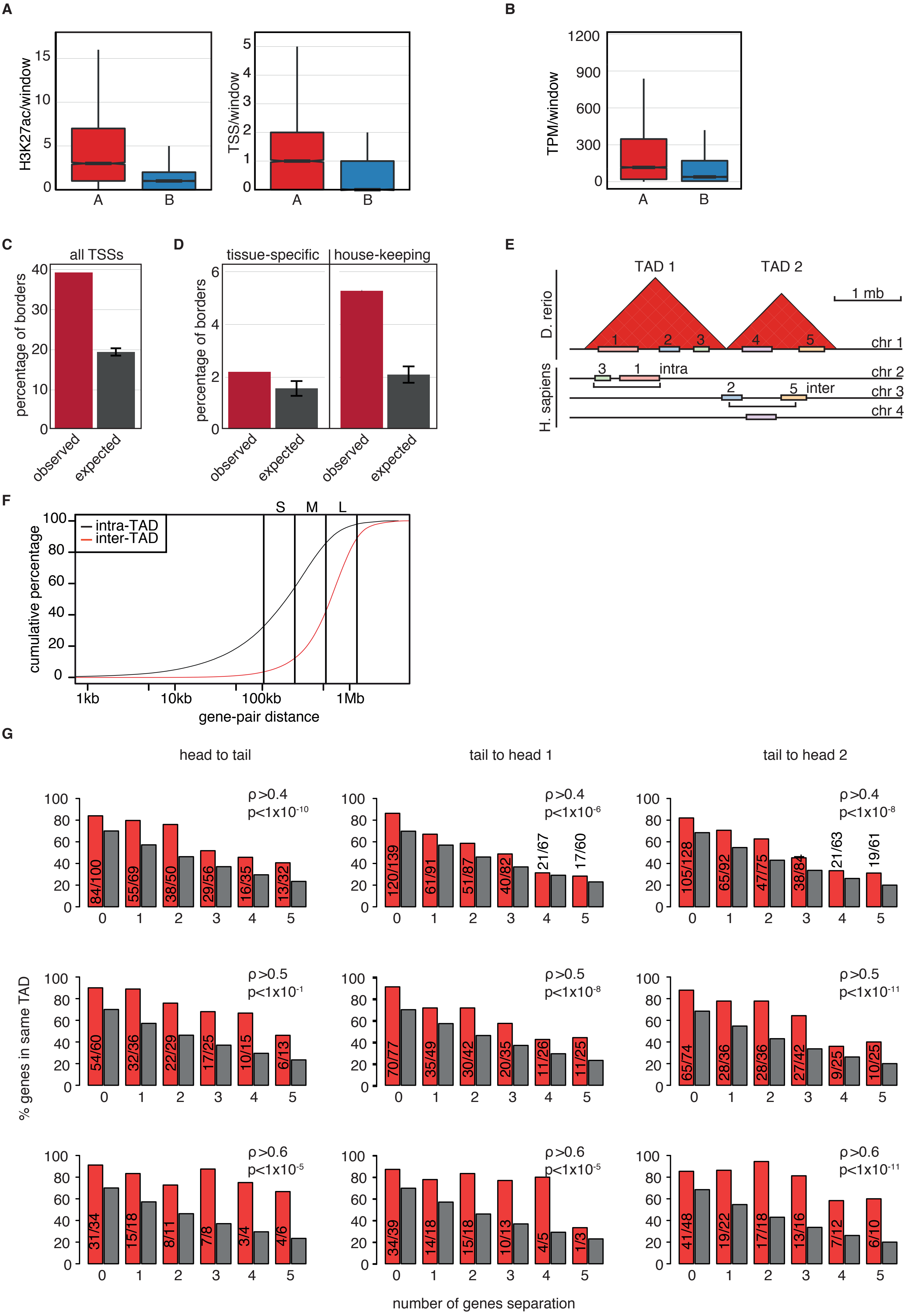

Figure S1. Zebrafish TADs recapitulate known features of TADs. Related to Figure 1
(A) Boxplot showing the distribution of H3K27ac ChIP-seq peaks and gene density in the A/B compartment, as indicated. The Wilcoxon rank sum test reveals significant difference between compartments for H3K27ac and gene density ($p=1.7x10^{-90}$ and $p=7.1x10^{-28}$, respectively). (B) Boxplot showing the transcripts per million of genes in the A/B compartment, as indicated. Wilcoxon rank sum test p-value reveals significant difference ($p=3.3x10^{-372}$). (C) Barplot showing the overlap of TSSs from all EnsEMBL genes with TAD borders. Confidence intervals were obtained by 1000 circular permutations of the TAD borders ($p<0.001$). (D) Barplot showing the overlap of house-keeping and tissue specific TSSs, as indicated, with TAD borders. Empirical p-values were obtained by 1000 circular permutations of the TAD borders. $p=0.018$ and $p<0.001$ for tissue specific and house keeping genes, respectively. (E) Schematic representation of the analysis performed in figure 1C shows two zebrafish TADs with five genes (top part) that have an ortholog in humans (lower part). Genes 1 and 3 form an example of a gene pair, which are present in zebrafish within the same TAD and are within 1MB distance in humans. This situation is classified as intra-TAD conservation. Gene pair 2 and 5 is within 1mb in the human genome, but present in neighboring TADs in zebrafish. This is classified as inter-TAD conservation. (F) Cumulative distributions of intra- and inter-TAD gene-pairs (black and red, resp.). The three size-ranges (S, M and L) are used to stratify the conservation-analyses to overcome differences in numbers of intra- and inter-TAD gene-pairs. (G) Barplots showing the enrichment of correlated genes, as indicated, based on three different Tomo-Seq datasets (red bars) within TADs as compared to all genes (grey bars). Analysis similar to Figure 1E.

Supplemental figure 2

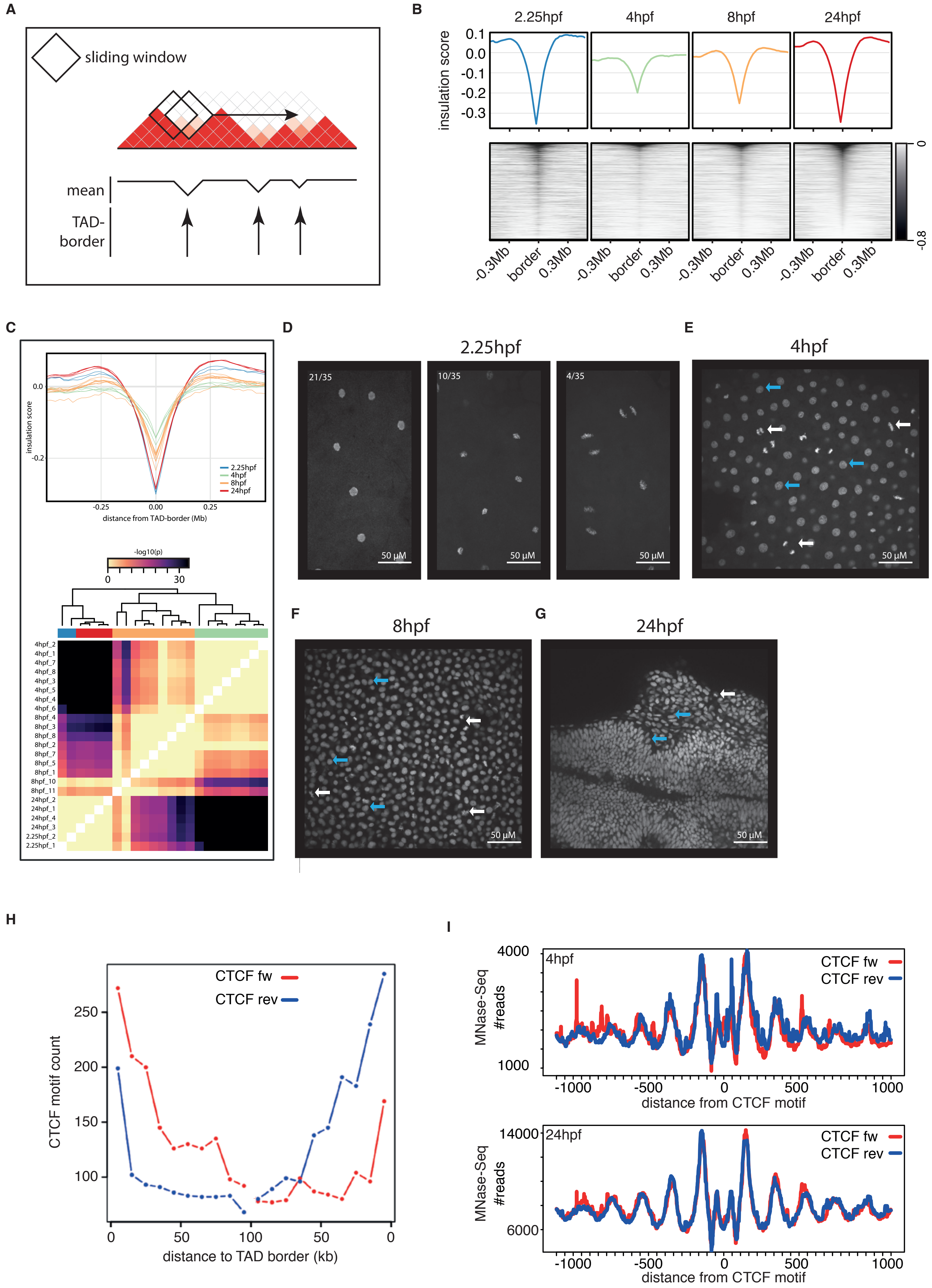

Figure S2 Hi-C analysis shows loss and reestablishment of chromatin architecture throughout development. Related to Figure 2.
(A) Schematic explanation of the insulation score. By sliding a square along the diagonal of a Hi-C matrix (shown here horizontally) the insulation score is calculated. At TAD borders there will be a segregation of interactions leading to a reduction in the score. TAD borders therefore show a local minimum in the insulation score. (B) Average insulation scores around 24hpf TAD borders throughout zebrafish development, as indicated (top panel). Bottom panel shows the insulation scores for the individual TAD borders for all four Hi-C datasets in a heatmap. (C) Upper panel shows the average insulation scores aligned to TAD borders for all the replicate Hi-C templates (2.25hpf, n = 2; 4hpf, n = 8; 8hpf, n = 9; 24hpf, n = 4). Bottom panel shows a heatmap quantifying the (dis)similarity of Hi-C templates of different timepoints. Pairwise difference between all templates is visualized as the Wilcoxon rank-sum test p-value of the insulation scores at the TAD borders. Hierarchical clustering shows the clustering of templates from the same timepoint. (D-G) Representative images of DAPI stained zebrafish embryos at indicated developmental stages. At 2.25hpf the embryos divide synchronously and the number of embryos showing a certain nuclear staining is indicated in the top left of the image. White arrows indicate nuclei in metaphase and blue arrows indicate nuclei in other cell cycle stages. (H) Motif count and orientation of CTCF binding sites inferred from 4hpf ATAC-seq relative to TAD borders. (I) MNase-seq read density surrounding ATAC-seq inferred CTCF binding sites at 4hpf (top panel) and 24hpf (bottom panel). CTCF binding sites are separated on their orientation, as indicated.

Supplemental figure 3

A

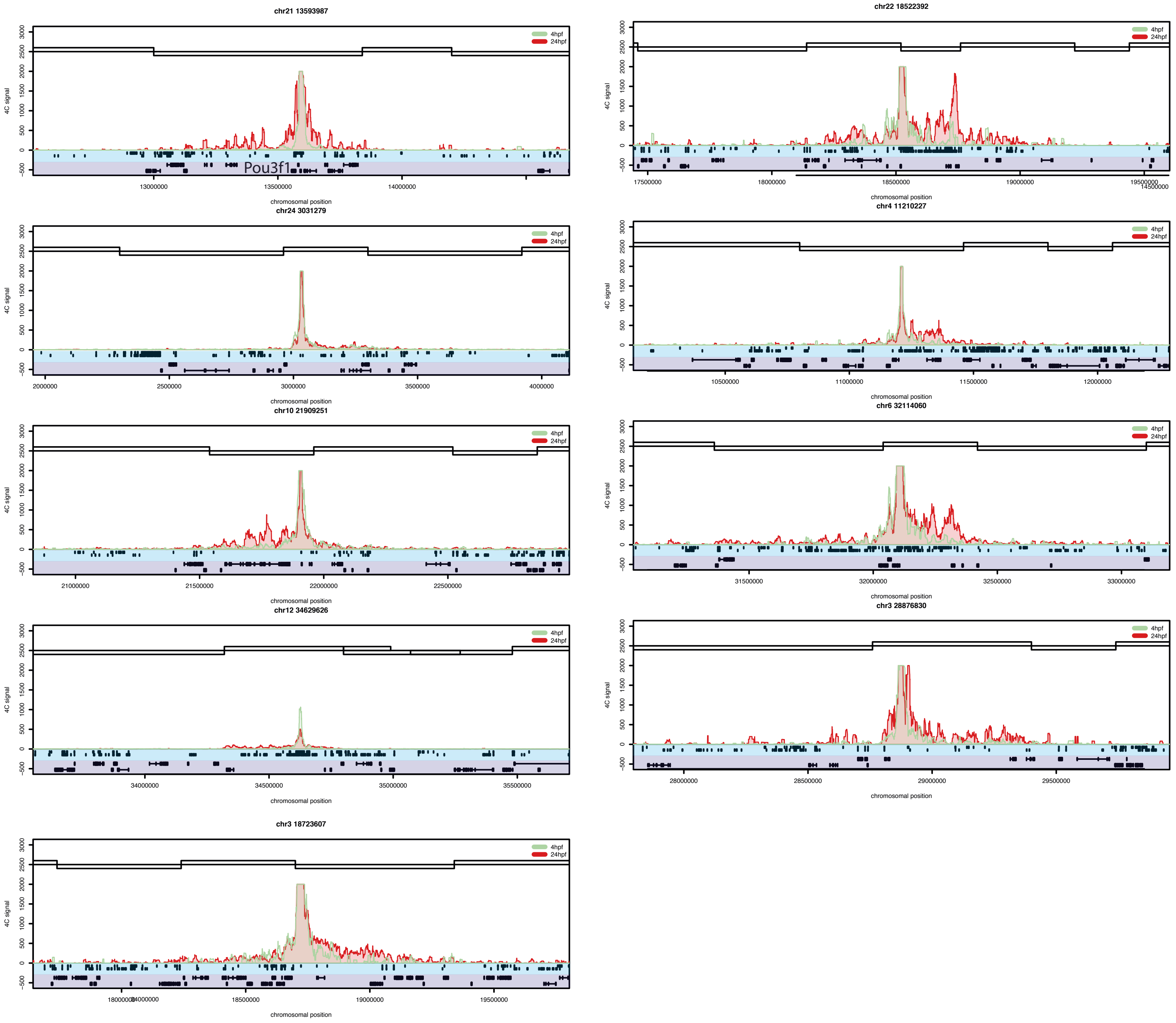

Figure S3 4C analysis shows lower intra-TAD contact frequency at 4hpf compared to 24hpf. Related to Figure 2
(A) 4C-seq plots show the contact frequency at 4hpf and 24hpf (n =1 is for both). Visualization is the same as in Figure 2E.

Supplemental figure 4

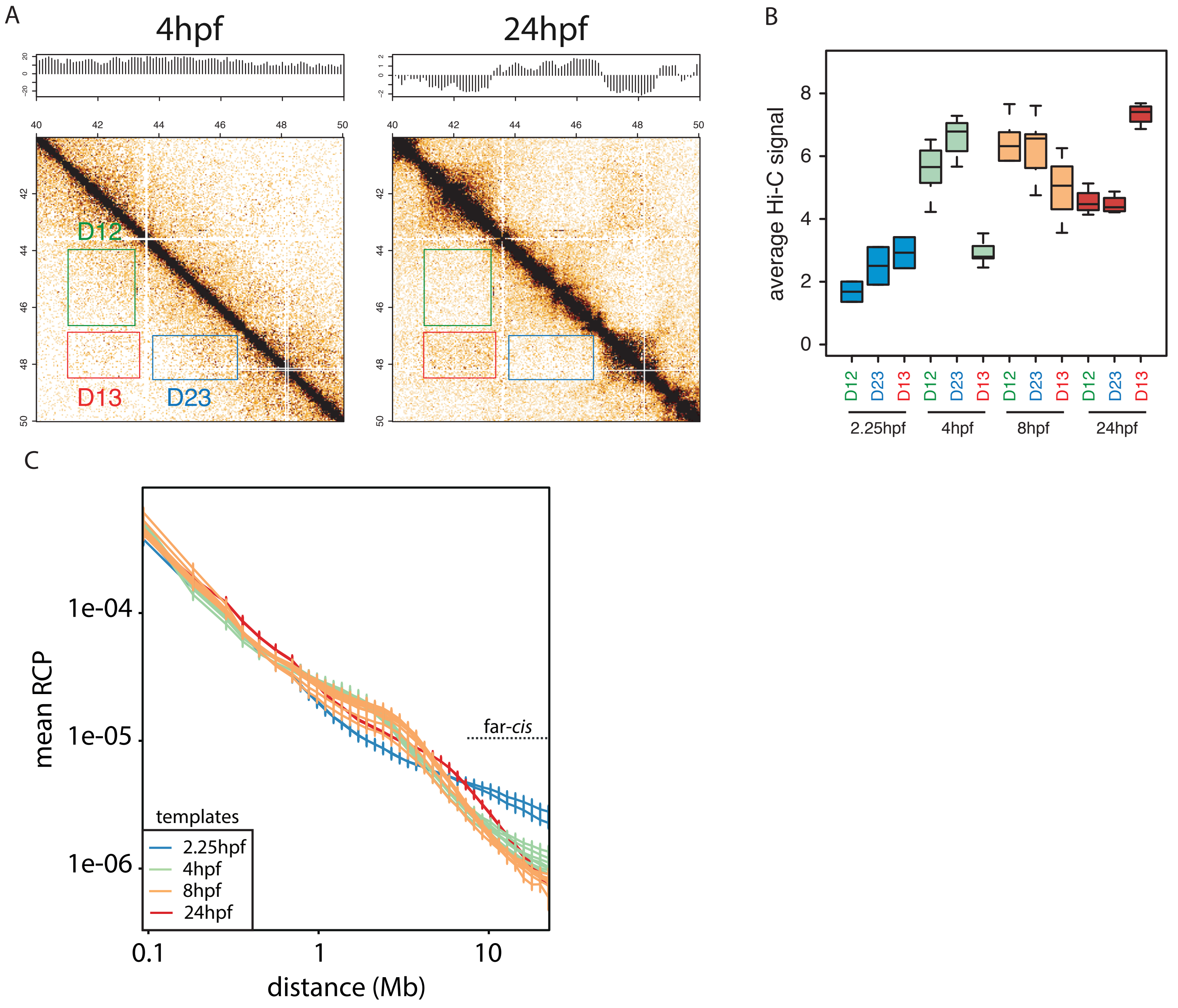

Figure S4. Differential compartmentalization is confirmed in individual replicates. Related to Figure 4.
(A) A Hi-C matrix of 4hpf (left) and 24hpf (right), annotated with their respective compartment-scores (top). The regions D12 and D23 are inter-compartmental and D13 is intra-compartmental. (B) Quantification of average Hi-C signal in intra- and inter-compartment regions, performed for all replicates at different time points (2.25hpf, n = 2; 4hpf, n = 8; 8hpf, n = 9; 24hpf, n = 4). (C) Relative contact probability plot with lines for every replicate (2.25hpf, n = 2; 4hpf, n = 8; 8hpf, n = 9; 24hpf, n = 4). Vertical lines represent SEM values per distance. (D) PE-Scan analysis using the 4hpf Hi-C data of all intra-chromosomal interactions between super-enhancers called at 8hpf.
